# Drug-Path: a database for drug-induced pathways

**DOI:** 10.1093/database/bav061

**Published:** 2015-06-30

**Authors:** Hui Zeng, Chengxiang Qiu, Qinghua Cui

**Affiliations:** ^1^Department of Cardiology, Peking University Third Hospital, Beijing 100191, China and; ^2^Department of Biomedical Informatics, MOE Key Lab of Cardiovascular Sciences, School of Basic Medical Sciences, Peking University, Beijing 100191, China

## Abstract

Some databases for drug-associated pathways have been built and are publicly available. However, the pathways curated in most of these databases are drug-action or drug-metabolism pathways. In recent years, high-throughput technologies such as microarray and RNA-sequencing have produced lots of drug-induced gene expression profiles. Interestingly, drug-induced gene expression profile frequently show distinct patterns, indicating that drugs normally induce the activation or repression of distinct pathways. Therefore, these pathways contribute to study the mechanisms of drugs and drug-repurposing. Here, we present Drug-Path, a database of drug-induced pathways, which was generated by KEGG pathway enrichment analysis for drug-induced upregulated genes and downregulated genes based on drug-induced gene expression datasets in Connectivity Map. Drug-Path provides user-friendly interfaces to retrieve, visualize and download the drug-induced pathway data in the database. In addition, the genes deregulated by a given drug are highlighted in the pathways. All data were organized using SQLite. The web site was implemented using Django, a Python web framework. Finally, we believe that this database will be useful for related researches.

**Database URL:** http://www.cuilab.cn/drugpath

## Introduction

The pathways of drugs are important information for not only understanding the mechanisms of drug action and metabolism but also for drug repositioning (1), which finds new therapeutic indications for approved drugs and experimental drugs that fail approval in their initial indication. Therefore, databases collecting drug pathways are increasingly important. Currently, several databases have included drug pathways, such as DrugBank (2, 3), KEGG (4, 5) and SMPDB (1, 6). These databases are useful to drug-related studies. However, most of the drug pathways in the above databases are pathways for drug action and drug metabolism (1). In addition, these pathways are greatly dependent on validated drug-binding targets, which are normally limited in number. Actually, besides its targets, one drug can induce expression changes of a number of genes, and thus can deregulate a number of pathways (7). In recent years, high-throughput technologies such as microarray and RNA-sequencing have produced a lot of drug-induced gene expression profiles. Among which, the Connectivity Map (CMap) (7) database contained gene expression profiles induced by more than 1000 drugs. These profiles play critical roles in a number of important topics, such as discovery of mode of drug action (8), drug-repurposing (9, 10), prediction of drug combinations (11) and construction of disease-drug network (12). As a result, molecular activity generated from global gene expression profiling now emerges as a promising resource for drug research (7). As one of the most comprehensive resources for drug-induced gene expression profiles, CMap provides us a chance to systematically identify the drug-induced deregulated genes and then identify the drug-induced pathways, which are believed to be important supplement to traditional drug pathways for drug action and drug metabolism. However, currently such a database is not available.

In this study, we present Drug-Path, a database of drug-induced pathways. By first identifying upregulated and downregulated genes of drug-induced gene expression profiles and then performing KEGG pathway enrichment analysis, we identified the pathways affected by drugs, which we called drug-induced pathways. All data were organized in the ‘Drug-Path’ database using SQLite, a lightweight database management system. The website is presented using Django, a Pythonweb framework. Drug-Path provides interfaces to freely retrieve, visualize and download the drug-induced pathways. Drug-Path is freely available at http://www.cuilab.cn/drugpath.

## Methods

### Data content

We obtained the drug-induced deregulated genes from the CMap database (http://www.broadinstitute.org/cmap/), which includes more than 1000 drugs. We obtained 94 human KEGG pathways from the KEGG database (http://www.kegg.jp/).

### Enrichment analysis

From the ‘amplitudeMatrix’ file derived from the CMap database, we selected 1294 drugs which were used to perform transcriptional microarray experiments on MCF7 cell lines. Each gene impacted by a given drug instance with fold-change value >2 or <0.5 was included into either the upregulated gene set or downregulated gene set of this drug instance, respectively. As each drug often has more than one instance, we combined all the results to generate the upregulated genes and downregulated genes for each drug.

**Figure 1.**
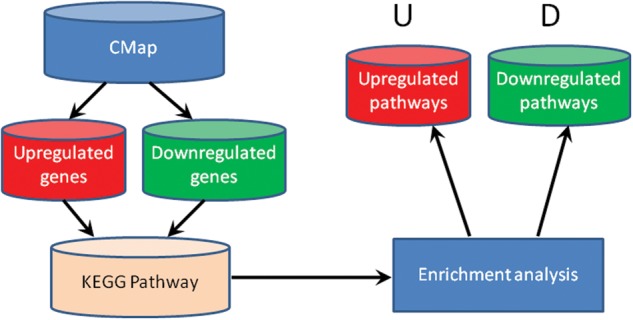
The flowchart of Drug-Path.

For one drug, using Fisher’s exact test we performed enrichment analysis of the upregulated genes and the downregulated genes induced by the drug in KEGG pathways, respectively. The *P* value was corrected by FDR as well. Then we obtained the upregulated pathways (U) and downregulated pathways (D) of this drug. The flowchart is as shown in Figure 1. We applied this pipeline to all the drugs in CMap.

### Database organization and web interface

All the data were stored and managed in a database organized by SQLite, a lightweight database management system. All data can be freely downloaded from the website. A user-friendly web interface has been designed and implemented for ‘Drug-Path’ using Django, a Pythonweb framework. Drug-Path provides interfaces to freely retrieve, visualize and download drug-induced pathways. The web client is developed independently of operating system. Here, we used the SVG file with different colors for each highlighted candidate genes to map into the given pathway.

### Data search

There are two options for users to search the pathways induced by a drug. First, the ‘quick search box’ can be found at the search page by inputting the name of a drug. Second, when users click ‘Click here to show the full drug list’, the list of all the drugs will be shown. Then when one drug was clicked, its name will be automatically inputted into the search box. For both options, when one drug was inputted, the induced pathways will be listed in the page when ‘Click to Analysis’ was clicked.

For example, we identified that the most significant pathways potentially induced by oligomycin were ‘olfactory transduction’ and ‘systemic lupus erythematosus’ through its upregulated genes and downregulated genes, respectively. Each row in the search result table included eight items: drug name, KEGG pathway ID, KEGG pathway name, the amount of genes in the pathway, the amount of overlaps, up-regulated or down-regulated genes, *P* value and the corrected FDR value. By clicking ‘olfactory transduction’, you will get the pathway figure including seven highlighted genes impacted by oligomycin in a new webpage. In the pathway figure, the upregulated genes are in yellow color and the downregulated genes are in red color. From the download page, you can get the whole dataset including the search results and the overlap genes characterized by Entrez Gene IDs.

## Results and Discussion

**Figure 2.**
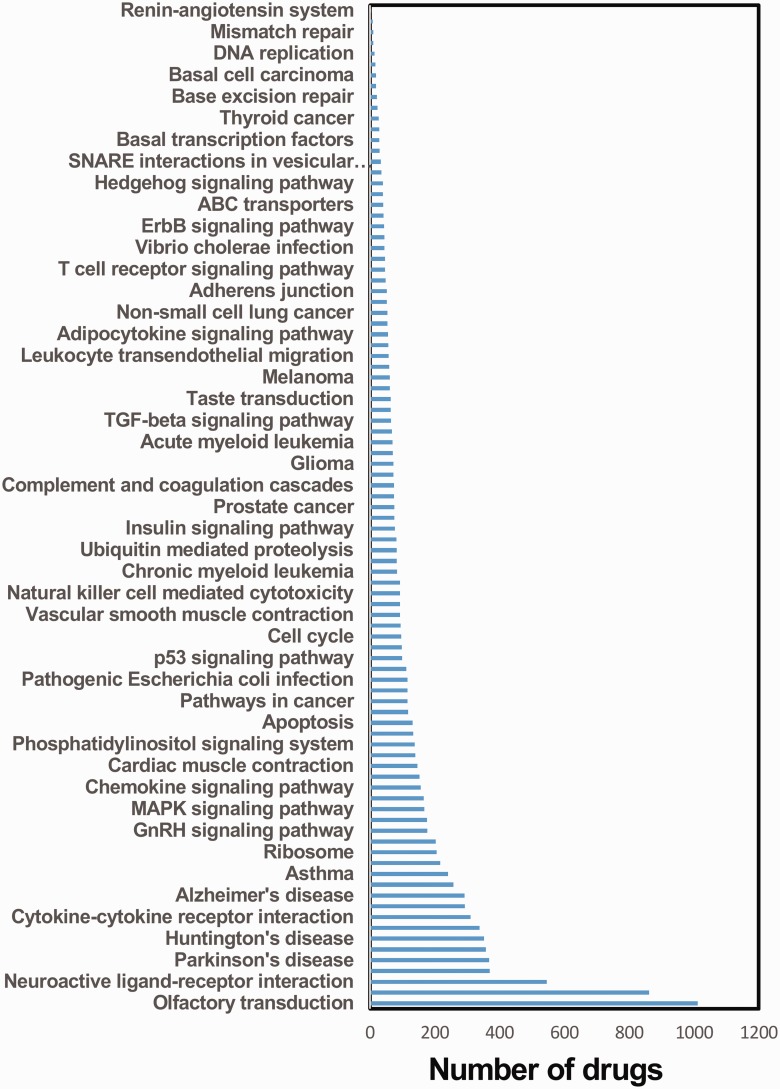
The number of drugs inducing each given pathway.

In this study, we developed a database for drug-induced pathways, Drug-Path, which contained 243 272 drug-pathway pairs, including 1294 drugs and 94 KEGG pathways. Each drug has two main categories of induced pathways, the pathways upregulated by the drug and the pathways downregulated by the drug. With the *P*-value <0.05 as a threshold, we identified the potential induced pathways for each given drug. The drug with the maximum number of pathways is daunorubicin, and it can impact as many as 40 pathways. For the pathways, we summarized the number of drugs that induced the given pathway (Figure 2). The pathway with the maximum number of drugs is olfactory transduction, which is induced by more than 1000 drugs. We believe that Drug-Path could serve as a valuable resource for unveiling the pathways induced by drugs.

Although Drug-Path shows value in mining drug information, limitations exist in the current method. One limitation is that the number of drugs is 1294, which only covers a small part of all drugs. Mining the drug-induced gene profile in other databases, such as GEO, will be an important supplement to the current database. Second, the current method only integrated KEGG pathways. It is also important to integrate other gene annotations, such as BioCarta signaling pathways and gene ontology. Therefore, in the future we would improve the Drug-Path by integrating more gene annotations. Finally, although problems exist in the current method, we believed it present a simple and valuable resource for understanding drug mechanisms.

## Funding

This study was supported by the Natural Science Foundation of China (No. 91339106). Funding for open access charge: the Natural Science Foundation of China (No. 91339106).

*Conflict of interest.* None declared.
